# Elimination of a Postoperative Brace Does Not Increase Complications Following Hip Arthroscopy

**DOI:** 10.7759/cureus.40321

**Published:** 2023-06-12

**Authors:** Andrea H Johnson, Erica Richardson, Brook Fowler, Michaline West, Justin J Turcotte, Benjamin M Petre

**Affiliations:** 1 Orthopedics, Anne Arundel Medical Center, Annapolis, USA; 2 Physical Therapy, Anne Arundel Medical Center, Annapolis, USA; 3 Clinical Research, Anne Arundel Medical Center, Annapolis, USA; 4 Orthopedic Research, Anne Arundel Medical Center, Annapolis, USA; 5 Orthopedic Surgery, Anne Arundel Medical Center, Annapolis, USA

**Keywords:** femoro-acetabular impingement, postoperative rehabilitation, postoperative complications, postoperative brace, hip arthroscopy

## Abstract

Background

The practice of routine postoperative bracing to limit abduction and internal rotation, along with weight-bearing restrictions after hip arthroscopy (HA), varies significantly among surgeons. It is unclear whether the use of a postoperative brace improves short-term outcomes in patients undergoing HA. The purpose of this study was to determine the differences in patient outcomes before and after eliminating routine usage of a postoperative brace.

Methods

A retrospective review was conducted of 176 adult patients undergoing HA by a single, high-volume surgeon. The no-brace protocol was implemented in October 2020. The patients were divided into two groups: pre-implementation (January-October 2020) and post-implementation (October 2020-April 2021). Twenty-three patients that used a brace during the post-implementation period were excluded. All patients had weight-bearing restrictions with crutches for three weeks postoperatively. The primary endpoint was any complication in the first six weeks postoperatively.

Results

There were no significant differences in demographics between groups, although the body mass index in the brace group was higher (28.1 vs. 26.4 kg/m^2^, p = 0.066) and the rate of additional procedures performed was higher in the brace group (55.2% vs. 40.1%, p = 0.056). There was no significant difference in postoperative outcomes between groups when looking at 90-day emergency department visits (1.7% vs. 0%, p = 0.548), complications at two (1.7% vs. 1.7%, p = 1.000) and six weeks (0% vs. 1.7%, p = 0.341) postoperatively, all complications in the first six weeks (1.7% vs. 1.7%, p = 1.000), and continued pain at six weeks (10.3% vs. 16.7%, p = 0.238).

Conclusion

The brace and no-brace groups were similar demographically. Patients undergoing HA with no brace and crutches experienced no significant differences in pain or complications in comparison to those receiving a traditional bracing protocol. Routine use of a postoperative brace may not be necessary in this population.

## Introduction

The utilization of hip arthroscopy (HA) for the treatment of femoroacetabular impingement syndrome (FAIS) has increased dramatically in the last 15 years. From 2006 to 2010, a 600% increase in procedure volume was observed in the American Board of Orthopedic Surgery (ABOS) Part II examinees, and the overall incidence increased from 3.6 per 100,000 in 2005 to 16.7 per 100,000 in 2013 [1,2]. FAIS is a fairly recently described phenomenon in which the femoral head or neck abuts the acetabular rim leading to damage to the articular cartilage and predisposing the patient to hip osteoarthritis [3-5]. There are three types of FAIS currently described in the literature: cam impingement, pincer impingement, and mixed impingement [6]. Cam impingement, which is more common in young and active male patients, results from a morphologic variation of the femoral head and results in a nonspherical femoral head abutting the acetabular rim during normal motion causing tearing or avulsion of the labrum and chondral damage in the anterosuperior area of the acetabulum [7,8]. Pincer impingement, which is more commonly seen in active middle-aged women, is caused by various abnormalities in the acetabulum, including acetabular retroversion, coxa profunda, and protrusio acetabuli, and results in tearing and/or degenerative changes in the labrum and chondral injury of the posteroinferior acetabulum [7,8]. Mixed impingement has features of both cam and pincer morphologies [9].

Rehabilitation protocols after HA, specifically with postoperative bracing and weight-bearing restrictions, are subject to significant variation even among high-volume HA surgeons [10]. The purpose of the hip brace is to limit flexion of the hip to 90°, limit hip abduction to 45°, and limit extension to 0° in order to protect the surgical repair [11]. There are no definitive studies in the literature regarding the effectiveness of postoperative bracing and whether it reduces pain or postoperative complications in this patient population. The brace itself is bulky and cumbersome and also costly, with a current estimated cost of $150-1400 USD. The purpose of this study is to evaluate patients undergoing HA for FAIS before and after the implementation of a no-brace protocol by examining emergency department returns, two- and six-week postoperative complications, and continued pain at six weeks following surgery. We hypothesize that patients will have similar outcomes before and after the implementation of a no-brace protocol in this institution.

This article was presented as a poster at the American Academy of Orthopedic Surgeons Annual Meeting March 23-25, 2022.

## Materials and methods

Study population and setting

This study was deemed institutional review board exempt by the institution’s clinical research committee. A retrospective observational study of patients undergoing HA for FAIS was performed in order to determine noninferiority. All surgeries were performed by a single surgeon in a single hospital-based outpatient surgery center from January 2019 to April 2021. All patients underwent arthroscopic surgery for a cam or pincer lesion and were surgically treated with bony resection and repair or reconstruction of the labrum, along with any other associated procedures including iliotibial band release or lengthening, gluteus medius repair, trochanteric bursectomy, psoas debridement or lengthening, and synovectomy.

Perioperative protocol

**Figure 1:**
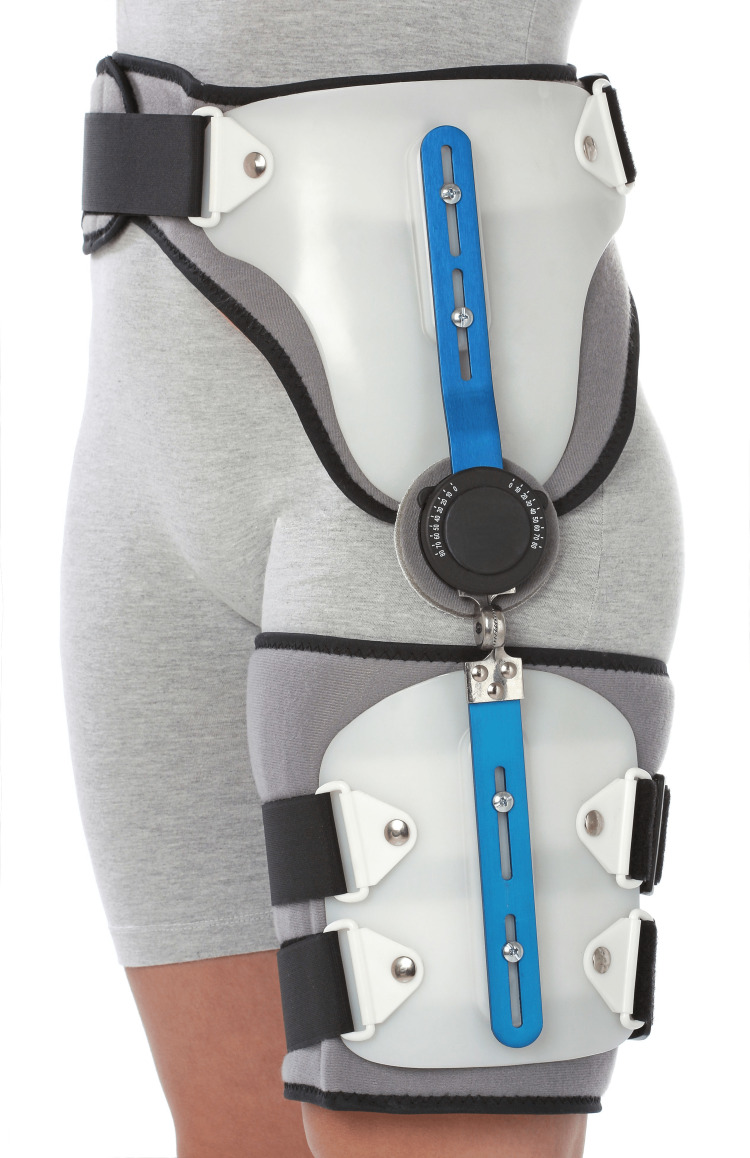
Postoperative hip range of motion brace Stock photo. Downloaded from https://www.shutterstock.com/image-photo/woman-hip-splint-over-white-39150331. Accessed June 7, 2023. Used with permission.

All surgeries were performed on an outpatient basis under general anesthesia; regional anesthesia for postoperative pain control was used at the discretion of the surgeon and anesthesiologist. All patients used the same rehabilitation protocols with the exception of brace usage. Prior to October 2020, all patients were fitted with a hip brace for use while walking, which limits hip flexion to 90° (Figure 1).

After October 2020, only select high-risk patients used the brace, and these patients were excluded from our analysis. All patients began a supervised rehabilitation program during postoperative week one. Initial range of motion (ROM) restrictions during physical therapy are hip flexion from 0° to 120 for zero to three weeks, abduction from 0° to 45° for zero to three weeks, and no external rotation for two weeks. Strength and ROM with restrictions are progressed per protocol. All patients are toe-touch weight-bearing with crutches from zero to three weeks, then progress to weight-bearing as tolerated. 

Power analysis

An a priori power analysis was performed in order to determine the target sample size for this noninferiority study. Using a 2:1 control to experimental ratio, it was determined that sample sizes of 138 control and 69 experimental cases should be targeted to achieve 80% power to detect a 10% absolute difference in complication rates between groups using a one-sided chi-square test at α = 0.05. The 10% noninferiority margin was selected in alignment with previous orthopedic studies [12].

Data collection and analysis

Demographics, comorbidities, and surgical details were manually recorded from the electronic medical record (EMR). The primary endpoint of the study was complications within the first six weeks postoperatively. This was broken down into any complication at two weeks postoperatively, any complication at six weeks postoperatively, and continued pain at six weeks postoperatively. Continued pain at six weeks postoperatively was defined as patient-reported pain at the six-week postoperative visit that was documented in the provider note. Univariate statistics (two-sided independent samples t-tests, chi-square tests, and Fisher’s exact tests) were performed to evaluate differences in demographics, comorbidities, and operative characteristics between patients who used a brace postoperatively and those that did not. All statistical analysis was performed in IBM SPSS Statistics, version 27.0 (IBM Corp., Armonk, NY), and statistical significance was assessed at α = 0.05.

## Results

One hundred and seventy-six patients were included in this study: 116 prior to October 2020 who used a brace postoperatively and 60 from October 2020 who did not use a brace postoperatively. Table 1 examines the demographic and surgical details between groups.

**Table 1:** Patient demographics and surgery details All data presented as mean ± SD or n (%); p-values <0.05 in bold *Fisher’s exact test **Additional procedures besides cam resection, pincer resection, labral repair, or labral reconstruction ASA, American Society of Anesthesiologists Score

Variable	No brace (N = 60)	Brace (N = 116)	p-value
Age, years	39.9 ± 14.8	39.8 ± 14.8	0.981
Body mass index, kg/m^2^	26.4 ± 6.4	28.1 ± 5.6	0.066
Female	42 (70.0)	73 (62.9)	0.350
ASA ≥ 3	8 (13.3)	17 (14.7)	0.812
Revision hip arthroscopy	3 (5.0)	8 (6.9)	0.751*
Additional procedures performed**	24 (40.0)	64 (55.2)	0.056

There were no significant differences between patients who used a brace postoperatively and those that did not; body mass index (BMI) was higher in the brace group (28.1 vs. 26.4 kg/m^2^, p = 0.066), and the rate of concomitant procedures performed was higher in the brace group (55.2% vs. 40.0%, p = 0.056). Concomitant procedures include any procedure other than cam resection, pincer resection, labral repair, and labral reconstruction.

Table 2 examines differences in postoperative outcomes between patients that used a brace postoperatively and those that did not.

**Table 2:** Postoperative outcomes All data presented as n (%); p-values <0.05 in bold *Fisher’s exact test **Excluding continued pain

Variable	No brace (N = 60)	Brace (N = 116)	p-value
30-day return to the operating room	0 (0.0)	0 (0.0)	n/a
90-day return to the emergency department	0 (0.0)	2 (1.7)	0.548*
Any complication within six weeks**	1 (1.7)	2 (1.7)	1.000*
Two-week complication	1 (1.7)	2 (1.7)	1.000*
Six-week complication**	1 (1.7)	0 (0.0)	0.341*
Continued pain at six weeks	10 (16.7)	12 (10.3)	0.238*

There were no significant differences between groups when examining 90-day emergency room visits (1.7% vs. 0%, p = 0.548), any complication within six weeks other than continued pain (1.7% vs. 1.7%, p = 1.000), complications in the first two weeks (1.7% vs. 1.7%, p = 1.000), any complication from three to six weeks (0% vs. 1.7%, p = 0.341), and continued pain at six weeks (10.3% vs. 16.7%, p = 0.238).

## Discussion

There was no significant difference in this population between patients that used a postoperative hip brace and those that did not. The groups were similar demographically, with no significant differences in age, BMI, sex, or American Society of Anesthesiologist score and also no significant difference in the rate of revision HA or concomitant procedures performed. There were also no significant differences in complication rate at two or six weeks postoperatively and no differences in continued pain at six weeks. Based on these findings, we suggest that routine use of hip bracing following HA for FAIS can be discontinued.

There are currently no published prospective or retrospective studies on the effect on patient outcomes and complications of postoperative hip bracing after HA in FAIS patients. There are a number of published surveys of HA surgeons on their practices and recommendations for intra- and postoperative protocols, and there is wide variability in the use of hip bracing. Gupta et al. surveyed 27 high-volume HA surgeons in the United States and found that 59.2% of these surgeons prescribed a postoperative hip brace for some or all of their patients [10]. A similar survey by Smith et al. of 75 international HA surgeons found that 48% of these surgeons reported prescribing a postoperative brace some or all of the time [13]. A more recent study by Bolia et al., which compared expert (>500 cases/lifetime) and nonexpert (≤500 cases/lifetime) HA surgeons, found a much lower rate of postoperative brace usage, with 40% of expert surgeons and 28% of nonexpert surgeons utilizing a brace during the postoperative period [14]. Based on these three published surveys, it appears that postoperative bracing is becoming less common over time.

Postoperative hip bracing contributes to the overall cost of care of the patient undergoing HA; current retail prices for postoperative hip braces range from $150 to $600 USD. With the increasing numbers of HA procedures being performed, this could be an economic concern with no clear benefit to the patient [2]. Due to the anatomic location of the hip joint, bracing this area is a challenge. Hip braces are typically bulky and cumbersome to use. There are no studies currently published in the literature examining patient satisfaction with postoperative hip bracing, although in a recent study by Newcomb et al., which looked at a less restrictive brace in patients being treated nonoperatively for FAIS, it was found that all patients included in the study reported one or more adverse event related to brace use [15].

This study does have a number of limitations. Firstly, it is a single institution, single surgeon observational cohort study, and it is possible that our population of patients is not representative of the broader population. Second, although we performed an a priori power analysis in order to determine the sample size needed for this study, based on the results, the study did not reach the targeted 80% power to identify differences between groups. With the exception of continued pain at six weeks, the overall complication rate in both groups was <2%, well below the 10% difference used to calculate a priori power. Although the rate of continued pain at six weeks was over 5%, the absolute difference between groups was only 6.4%. Using the data from this study for the rate of continued pain at six weeks, we performed a second power analysis and determined that, in order to prove a significant difference in the rate of patients experiencing continued pain at six weeks, we would need a brace group and a no-brace group of 444 patients each in order to reach 80% power. Based on the current rate of HA at any single institution, this would optimally be achieved in a multi-site trial, though this is likely not practical. Therefore, the opportunity exists to repeat our study at other institutions in order to compare results and confirm our findings. Despite these shortcomings, we do feel this study does contribute significant value to the literature as very few studies have been published that examine the effect of postoperative bracing on patient outcomes following HA. We did identify one actively enrolling clinical trial examining a similar question [16]; additional research is needed to investigate the effect of postoperative bracing on patient-reported outcomes and functional outcomes and also the cost-effectiveness of postoperative brace use.

## Conclusions

Patients undergoing HA after the implementation of a no-brace protocol at this institution experienced no significant differences in postoperative pain or complications in comparison to patients who underwent surgery with a traditional brace protocol. Routine use of a postoperative brace in patients undergoing HA for femoroacetabular impingement may not be necessary. Further study is required to validate these findings and determine the optimal protocol for this patient population.
